# The Genetic Structure of Wild *Orobanche cumana* Wallr. (Orobanchaceae) Populations in Eastern Bulgaria Reflects Introgressions from Weedy Populations

**DOI:** 10.1155/2014/150432

**Published:** 2014-07-20

**Authors:** Rocío Pineda-Martos, Antonio J. Pujadas-Salvà, José M. Fernández-Martínez, Kiril Stoyanov, Leonardo Velasco, Begoña Pérez-Vich

**Affiliations:** ^1^Institute for Sustainable Agriculture (IAS-CSIC), Finca Alameda del Obispo, Avenida Menéndez-Pidal s/n, 14004 Córdoba, Spain; ^2^Department of Agricultural and Forestry Sciences and Resources, University of Córdoba, Campus de Rabanales, Edificio Celestino Mutis, Carretera de Madrid Km 396, 14071 Córdoba, Spain; ^3^Department of Botany, Agricultural University of Plovdiv, 12 Mendeleev, 4000 Plovdiv, Bulgaria

## Abstract

*Orobanche cumana* is a holoparasitic plant naturally distributed from central Asia to south-eastern Europe, where it parasitizes wild Asteraceae species. It is also an important parasitic weed of sunflower crops. The objective of this research was to investigate genetic diversity, population structure, and virulence on sunflower of *O. cumana* populations parasitizing wild plants in eastern Bulgaria. Fresh tissue of eight *O. cumana* populations and mature seeds of four of them were collected *in situ* on wild hosts. Genetic diversity and population structure were studied with SSR markers and compared to weedy populations. Two main gene pools were identified in Bulgarian populations, with most of the populations having intermediate characteristics. Cross-inoculation experiments revealed that *O. cumana* populations collected on wild species possessed similar ability to parasitize sunflower to those collected on sunflower. The results were explained on the basis of an effective genetic exchange between populations parasitizing sunflower crops and those parasitizing wild species. The occurrence of bidirectional gene flow may have an impact on wild populations, as new physiological races continuously emerge in weedy populations. Also, genetic variability of wild populations may favour the ability of weedy populations to overcome sunflower resistance mechanisms.

## 1. Background

Broomrapes (*Orobanche* spp. and* Phelipanche* spp.) are a group of around 170 holoparasitic plant species mainly distributed in the northern hemisphere. They do not have photosynthetic activity and entirely depend on a host plant for nutrition [1]. Even though most of the* Orobanche* spp. only parasitize wild plants, some of them have become noxious weeds on a variable range of cultivated hosts [2]. This is the case of* Orobanche cumana* Wallr. (sunflower broomrape), which is nowadays one of the most limiting factors for sunflower (*Helianthus annuus* L.) production in Europe and Asia [3].

*Orobanche cumana* is naturally distributed from central Asia to south-eastern Europe, where it parasitizes wild Asteraceae species, mainly* Artemisia* spp. [4]. Even though it has been considered by some authors as an intraspecific taxon of* Orobanche cernua* L. [5], its treatment as a separate species is nowadays widely accepted [2, 6, 7]. The Black Sea coast in eastern Bulgaria is one of the main natural distribution areas for* O. cumana*, where this species is mainly found parasitizing* Artemisia maritima* L. [8].

Though domesticated in eastern North America and widely used as a staple food in the pre-Columbian period [9], the transformation of sunflower into one of the major world oil crops started in Russia in the second half of the nineteenth century [10]. Plants of* O. cumana* parasitizing sunflower were observed for the first time in Russia in the 1890s [11]. In Bulgaria,* O. cumana* parasitization on sunflower was first detected in 1935 [12]. Currently,* O. cumana* is present in the main sunflower-producing countries around the world, particularly in Central and Eastern Europe, Spain, Turkey, Israel, Russia, Ukraine, Iran, Kazakhstan, and China [2]. Moreover, the parasite has spread to new areas in recent years [13, 14]. Broomrape seed transport has been suggested as one of the main factors in the dispersion of the infestation [15]. Broomrape seeds are extremely small (dust-like seeds), and individual plants can produce an impressive number that remain viable in the soil for up to 20 years, which are easily dispersed by water, wind, animals, humans, machinery, or though attachment to sunflower seeds [15, 16].

Unlike most weedy* Orobanche* spp., which have a broad range of host crops, weedy* O. cumana* only parasitizes sunflower [2]. The high host specificity of* O. cumana* is probably associated with the mode of inheritance of genetic resistance in sunflower. Whereas in most host crops genetic resistance to* Orobanche* spp. is horizontal, that is, polygenic and nonrace specific, resistance to* O. cumana* in sunflower is primarily vertical, that is, monogenic, dominant, and race specific [16]. The development of sunflower resistant cultivars has been paralleled by the appearance of* O. cumana* populations that overcame sunflower genetic resistance, a recurrent process that has continued until today [11]. Several physiological races of* O. cumana* have been reported. Vrânceanu et al. [17] identified races A through E using five sunflower differential lines carrying the dominant resistance genes* Or1* through* Or5*, respectively. More virulent races named as F, G, and H were later detected in the main sunflower cultivation areas of the Old World [3]. In Bulgaria, races D and E were predominant till few years ago [18], but a more virulent race G has become increasingly important in recent years [19].

There are few studies on genetic interactions between wild and weedy forms of parasitic plant species. Knowledge about such interactions is important because wild vegetation may play a role as reservoir of genetic diversity for overcoming genetic resistance mechanisms in the host crops [20, 21]. But on the other hand, evolution of virulence in weedy populations may also have an impact on the distribution of the species in the wild [22]. Botanga et al. [23] used seeds of eight populations of the parasitic weed* Striga asiatica* (L.) Kuntz collected on wild hosts to conduct infestation experiments on susceptible maize and sorghum cultivars. None of the populations parasitized on sorghum, whereas five out of the eight populations failed to parasitize on maize. The authors concluded the occurrence of local adaptation of the parasite to a host species as well as a high degree of host specialization. Similarly, Botanga and Timko [24, 25] reported the stratification by host preference of* Striga gesnerioides* (Willd.) Vatke genotypes parasitizing cowpea [*Vigna unguiculata *(L.) Walp.] and the wild legume* Indigofera hirsuta* L. Conversely, Olivier et al. [26], using isozyme loci, showed little genetic differentiation based on host specificity among* Striga hermonthica* (Del.) Benth. populations parasitizing sorghum, pearl millet, maize, and wild grasses. Similarly, Vaz Patto et al. [20] found low genetic differentiation between populations of* Orobanche foetida* Poir. collected on a wild host and a population growing on cultivated vetch (*Vicia sativa* L.) using AFLP analyses.

Studies on genetic diversity within and between* O. cumana* populations are scarce and focused on weedy populations collected on sunflower. Gagne et al. [27] studied genetic diversity in eight populations from several countries using RAPD markers. They identified large interpopulation and low intrapopulation genetic variation, concluding the existence of two main gene pools, one comprising populations from Eastern Europe and another one including populations from Southern Spain. Pineda-Martos et al. [28] identified two main gene pools for* O. cumana* in Spain, comprising populations from the Guadalquivir Valley (Southern Spain) and Cuenca Province (Central Spain), respectively. Both groups were genetically distant, but both intra- and interpopulation genetic variation were in general extremely low within each gene pool due probably to a founder effect. However, a reduced number of populations exhibited larger genetic diversity, which was attributed to the presence of individuals from both gene pools and the occurrence of crosses between them. Even though* O. cumana *is considered to be primarily a self-pollinated species [29], the occurrence of a certain rate of cross pollination has been experimentally demonstrated [30].

There is no information on the population structure of* O. cumana* populations parasitizing wild species and their genetic relationship with weedy populations in areas where they coexist. There is also no information on their virulence on sunflower. The objective of this research was to investigate the genetic diversity, population structure, and ability to parasitize sunflower of* O. cumana* populations growing on wild plants in the Black Sea coast of Bulgaria, as well as their relationship with weedy populations parasitizing sunflower.

## 2. Materials and Methods

### 2.1. *Orobanche cumana* Populations

**Figure 1:**
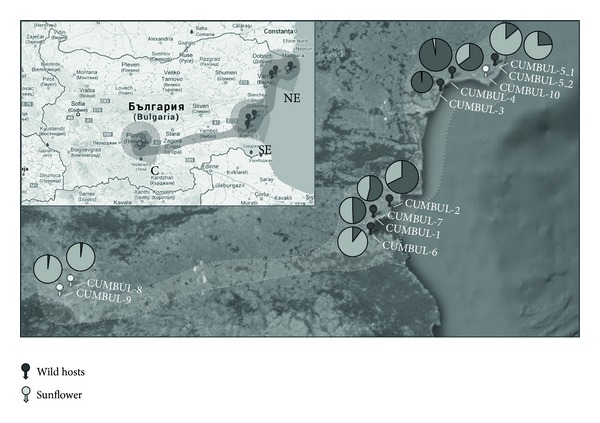
Geographical distribution of* Orobanche cumana* Bulgarian populations collected on wild and cultivated hosts (left side of the figure) and map of mean membership probabilities per population as obtained using Bayesian clustering analysis resulting from STRUCTURE at *K* = 2 (right side of the figure). Pie size is proportional to the size of each population.

**Figure 2:**
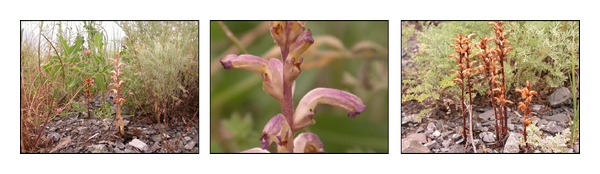
Details of population CUMBUL-1 of* Orobanche cumana* parasitizing* Artemisia maritima* in Burgas, Bulgaria.

Two field expeditions were conducted in July 2006 and June 2012 along the Black Sea coast of Bulgaria, where the distribution of* O. cumana* in the wild has been largely documented [8, 31–33], to collect fresh tissue and mature seeds of* O. cumana* populations parasitizing wild Asteraceae species. Six populations were located in both expeditions, one of them in both years (Table 1, Figure 1). Samples from the latter population were managed separately in the study to evaluate potential changes between both collection dates. Voucher specimens of the populations are housed in the herbarium of the University of Córdoba, Spain (herbarium code COA). Duplicated specimens can also be found at the herbarium SOA (Agricultural University of Plovdiv, Bulgaria). Populations CUMBUL-1 (COA-45783 and COA-45784), CUMBUL-2 (COA-45789), CUMBUL-3 (COA-45790), CUMBUL-4 (COA-45785), CUMBUL-6 (COA-53262 and COA-54519), and CUMBUL-7 (COA-54510) were collected on* A. maritima* L. (Table 1).  Figure 2 shows details of population CUMBUL-1. Population CUMBUL-5 was found parasitizing* Anthemis arvensis *L.,* Chamaemelum nobile* (L.) All., and another species of the Asteraceae that could not be identified, though in the latter case only two plants were present and they were not collected. Plants collected on* A. arvensis* (CUMBUL-5_1; COA-45791) and* C. nobile* (CUMBUL-5_2; COA-45792) were analyzed separately to evaluate potential differences associated with the host plant. The populations were located at a distance of less than 3 km from agricultural fields. Fresh tissue (young stalks) from 6 to 30 individual plants (Table 1), depending on population size, was collected* in situ* for each population and kept under drying conditions in ziplock bags with silica gel for subsequent freezing at −80°C. Fresh tissue of three* O. cumana* populations parasitizing sunflower crops in two different areas of Bulgaria (Table 1) was collected in the 2012 expedition. Additionally, fresh tissue was also collected* in situ* from three populations parasitizing sunflower in two different areas of Spain in which contrasting gene pools have been identified [28], to be used as a control (Table 1).

Mature seeds were collected in bulk from 5 to 30 mature plants of populations CUMBUL-1, CUMBUL-2, CUMBUL-4, and CUMBUL-5_1. No mature plants were available at the time of the collection expeditions for the other populations, including the* O. cumana* populations parasitizing sunflower in Bulgaria. Alternatively, seeds from three populations of* O. cumana* collected in sunflower fields in Bulgaria (OC-9, OC-11, and OC-13) were used for virulence studies. Populations OC-9 and OC-13 were kindly provided by Professor Rossitza Batchvarova, AgroBioInstitute, Sofia, Bulgaria. Population OC-11 was collected by one of the authors (K. Stoyanov). Spanish race F population OC-88 was also used as a control for virulence studies.

### 2.2. DNA Extraction and SSR Analysis

Frozen tissue was lyophilized and ground to a fine powder. DNA was extracted from individual* O. cumana* plants using a modified version of the protocol described in Pérez-Vich et al. [34]. Microsatellite analyses were carried out as described in Pineda-Martos et al. [28], using the same set of fifteen high-quality, polymorphic SSR primer pairs (Table S1 in Supplementary Material available online at http://dx.doi.org/10.1155/2014/150432). Amplification products were resolved by electrophoresis on 3% Metaphor agarose (BMA, Rockland, ME, USA) gels in 1x TBE buffer at 100 V constant voltage, with SaveView Nucleic Acid Stain (NBS Biologicals Ltd., Huntingdon, UK) incorporated in the gels and visualized under UV light. A 100 bp DNA ladder (Solis BioDyne, Tartu, Estonia) was used as a standard molecular weight marker to get an approximate size of DNA fragments. Bands were scored manually with the aid of Quantity One 1-D Analysis Software (Bio-Rad Laboratories Inc., Hercules, CA, USA) at least twice independently for each population.

### 2.3. Molecular Data Analysis

#### 2.3.1. Genetic Diversity Analysis

For each SSR locus, the number of alleles (Na), observed and expected heterozygosity (Ho and He), and *F*_ST_ were calculated using GenAlEx ver. 6.5 [35]. Additionally, each locus was tested for departure from Hardy-Weinberg equilibrium (HWE) and linkage disequilibrium within each of the populations with Arlequin ver. 3.5.1.3 [36]. To characterize the genetic diversity of* O. cumana* populations collected on wild hosts and the control populations collected on sunflower, the percentage of polymorphic loci (*P*), the average observed number of alleles (Na), the number of different alleles with a frequency ≥ 5% (Na ≥ 5%), the number of effective alleles (Ne), the number of private alleles unique to a single population (Npa), the observed and expected heterozygosity (Ho and He), the Shannon's diversity index (I), and the fixation index (*F*_is_) were calculated for all loci at each population. All calculations were carried out using GenAlEx ver. 6.5. *F*_is_ was used to estimate the selfing rate (*S*) from *S* = 2*F*_is_/(1 + *F*_is_) [37]. As additional measures of intrapopulation diversity, the mean number of pairwise differences between individuals within each population, estimated as the mean number of differences between all pairs of SSR haplotypes in each population, and the genotypic richness (*R*), defined as (*G* − 1)/(*N* − 1), where *G* is the number of MLGs (the observed number of multilocus genotypes) and *N* is the number of samples per population, were determined using Arlequin ver. 3.5.1.3, and GenClone 2.0 [38], respectively.

#### 2.3.2. Genetic Differentiation Analysis

To evaluate genetic differentiation between populations, initial frequency-based analysis was carried out by calculating pairwise genetic distances between populations using the genetic distance coefficient *G*_ST_ as implemented in GenAlEx ver. 6.5 using 1000 random permutations to assess significance. Pairwise distance matrices were also calculated using GenAlEx ver. 6.5 with other frequency-based estimators of population structure for codominant data such as Nei's *G*_ST_, Nei's standardized *G*_ST_, Hedrick's standardized *G*_ST_, Hedrick's further standardized *G*_ST_ for small number of populations, and Jost's estimate of differentiation, following calculations detailed in [39]. The pairwise relationship between the genetic distance matrices was tested through a Mantel's test with 999 permutations. Since the different statistical measures were highly correlated (*r* > 0.94, *P* = 0.001 for all comparisons, excepting those including the Jost's estimate of differentiation in which *r* > 0.90, *P* = 0.001), only the results based on the genetic distance coefficient *G*_ST_ with the corrections of Nei and Chesser [40] and Nei [41] are presented. To assess genetic relationships among populations, the matrix of *G*_ST_ pairwise distances was used as input for a principal coordinates analysis (PCoA) using GenAlEx ver. 6.5. PCoA has the main advantage of not requiring strong assumptions about the underlying genetic model [42].

To identify genetically homogeneous groups (gene pools), Bayesian model-based clustering algorithms implemented in the software package STRUCTURE ver. 2.3.4 [43] were applied. Cluster grouping in STRUCTURE is based on iterative analysis using *K* number of groups previously defined by the user, with individuals in the sample being assigned probabilistically to one or several groups. The admixture model and the allele frequencies correlated model were used [44]. No prior information was used to define the clusters. For each value of *K* (from 1 to 14), 10 independent runs were made that were used to estimate the probability of the data Pr(*X*∣*K*). For each run, 1,000,000 Monte Carlo Markov chain (MCMC) iterations were carried out after a burn-in period of 200,000 steps. To detect the number of genetically homogeneous groups (*K*) that best fits the data, the STRUCTURE HARVESTER website [45], which implements the Evanno method [46], was used. The 10 runs from the most probable number of *K* groups were averaged applying the FullSearch algorithm provided in the CLUMPP ver. 1.1.2b software [47] and the output was entered into Distruct ver. 1.1 for display [48]. To explore the genetic structure further, the STRUCTURE analyses were also carried out only with the 11 Bulgarian populations, as described above. We also used the program InStruct [49] for analyzing population structure, since this program is an extension of STRUCTURE that does not assume Hardy-Weinberg equilibrium and can incorporate selfing in the model. In addition, it can estimate the level of selfing in each population. Five independent chains were run for each *K*. Each chain was run for 1,000,000 iteration steps, with a burn-in of 500,000, and a thinning of 10. Graphical representations of population assignments from InStruct were produced from the program Distruct ver. 1.1 [48].

Finally, an analysis of molecular variance (AMOVA) [50] within populations, among populations, and among population groups (based on* a priori* grouping variables such as wild or cultivated host or based on the gene pools determined with clustering methods) was carried out to determine the distribution of variation at different hierarchical levels. The variance components were tested statistically by nonparametric randomization tests using 1000 permutations. Fixation indices (*F*-statistics) were also estimated by AMOVA. All calculations were carried out with Arlequin ver. 3.5.1.3.

### 2.4. Parasitization Ability and Virulence on Sunflower

Mature seeds were collected for wild* O. cumana* populations CUMBUL-1, CUMBUL-2, CUMBUL-4, and CUMBUL-5_1. However, the amount of available seed was very low, which restricted the number of sunflower genotypes for virulence studies as well as the number of plants per genotype. Accordingly, their parasitization ability and virulence on sunflower was evaluated in two separated experiments. The first experiment was aimed at determining whether the populations had the ability to parasitize sunflower genotypes with no genetic resistance to weedy* O. cumana* physiological races. Two confectionery sunflower landraces, B117 and B206, with no known resistance to any* O. cumana* race were used. Both landraces were collected by L. Velasco in isolated vegetable patches in Valdepeñas (Jaén Province, Spain) and Quintana de la Serena (Badajoz Province, Spain), respectively.* Orobanche cumana* population OC-88 [with known virulence (race F)] was used as a positive control. In a second experiment, the virulence of the populations was tested on a set of sunflower lines with varying levels of genetic resistance to* O. cumana* physiological races. Jdanovski 8281 (J8281) is a line incorporating resistance gene* Or2* that confers resistance to* O. cumana* race B [17]. AC03-1589 is a line incorporating resistance gene* Or3* that confers resistance to* O. cumana* race C, kindly provided by Dr. Maria Păcureanu, National Agricultural Research and Development Institute, Fundulea, Romania. S1358 is a line incorporating resistance gene* Or4* that confers resistance to* O. cumana* race D [17]. P-1380 is a line containing the resistance gene* Or5*, which determines resistance to* O. cumana* race E [17]. P96 is a line with recessive resistance to* O. cumana* race F [34]. B117 with no known resistance to any* O. cumana* race was used as positive control. Populations OC-9, OC-11, OC-13, and OC-88 were used as controls. Because the amount of seed of populations CUMBUL-1, CUMBUL-4, and CUMBUL-5_1 was not enough for evaluating them on all sunflower lines of the second experiment, it was decided not to test them on line S1358.

Seeds of* O. cumana* populations were used to inoculate small pots 7 × 7 × 8 cm filled with a mixture of sand and peat (1 : 1 by vol). Twenty-five mg of* O. cumana* seeds per pot was used. The soil mixture containing* O. cumana* seeds was carefully mixed to obtain a homogeneously infested substrate. Seeds of sunflower cultivars were germinated on moistened filter paper in Petri dishes and two-day-old seedlings were planted in the pots inoculated with* O. cumana* seeds. Eight pots (replications) per combination of sunflower cultivar and* O. cumana* population were used. The plants were maintained in a growth chamber for 21 days at 25°C/20°C (day/night) with a 16 h photoperiod for incubation. After this time, the plants were transplanted to pots containing 3 L of an uninfested sand-silt-peat (2 : 1 : 1 by vol) soil mixture and maintained under open air conditions. The plants were watered as needed and were not fertilized. The number of* O. cumana* shoots per sunflower plant was counted at sunflower maturity. Differences between mean numbers of* O. cumana* shoots per plant for each* O. cumana* population and sunflower cultivar were analyzed through one way ANOVA and Tukey's range test using IBM SPSS Statistics version 19.

## 3. Results

### 3.1. Genetic Diversity and Population Structure

All SSR markers were polymorphic (Table S1). The total number of alleles scored was 38, ranging from 2 to 4 for each SSR locus. Allelic diversity was generally low for all fifteen SSR loci when considering the whole set of 260 individual* O. cumana* plants (Table S1). All the loci exhibited an important heterozygote deficiency (Table S1). A significant deviation (*P* < 0.05) from Hardy-Weinberg equilibrium was found for almost all loci when all samples were considered. Linkage disequilibrium was significant (*P* < 0.05) in 238 out of 430 paired loci comparisons when considering all the samples. It has been established that linkage disequilibrium is predicted to approach zero for an ideal population, in the absence of forces such as genetic drift, population mixing, mutation, natural selection, or inbreeding [51]. High linkage disequilibrium observed suggested the existence of some genetic structure, apart from other factors determining the organization of genetic variation in the studied populations, as it will be further discussed below.

Genetic diversity within each population, measured by the mean number of observed and effective alleles, the expected heterozygosity, and Shannon's diversity indexes, was in general low, and only one population (CUMBUL-4) contained a substantial number of private alleles (Table 2). As expected from previous studies, Spanish populations were characterized by extremely low level of intrapopulation genetic diversity due probably to a founder effect [28], with no polymorphic loci being detected in two out of the three populations (Table 2). In contrast, populations from Bulgaria exhibited higher diversity values, with the exception of population CUMBUL-3, which showed no polymorphic loci. However, it is important to note that this was the smallest population, in which only six individual plants could be collected. Amongst the other Bulgarian populations, the highest genetic diversity corresponded to the populations collected on wild hosts CUMBUL-2, CUMBUL-5_2, and CUMBUL-7, which showed He, I and pairwise difference (between individuals) values over 0.25, 0.4, and 3.5, respectively (Table 2). The lowest genetic diversity corresponded to populations CUMBUL-8 and CUMBUL-9, collected on sunflower, which showed He, I and pairwise difference (between individuals) values below 0.05, 0.1, and 0.5 respectively (Table 2). The other six populations, excluding CUMBUL-3, showed intermediate diversity values, ranging from 0.10 to 0.23 for He, from 0.18 to 0.35 for I, and from 1.8 to 3.1 for pairwise differences between individuals. The fixation index (*F*_is_) and selfing rate (*S*) values were high for the populations studied (Table 2).

**Figure 3:**
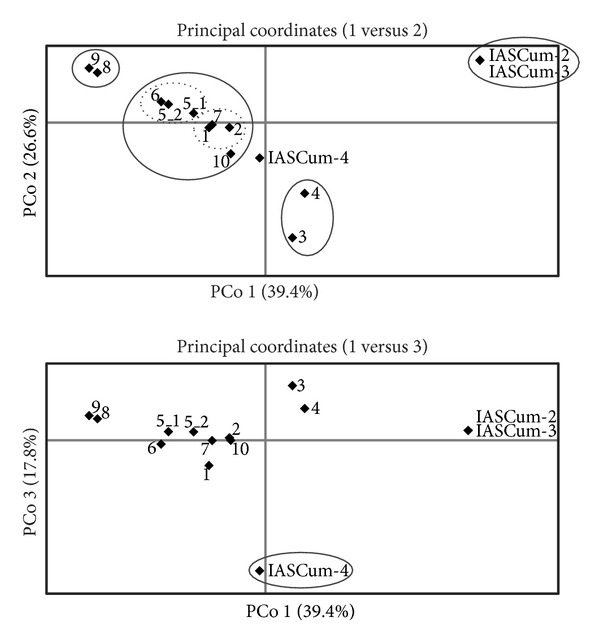
Principal coordinates analysis of pairwise genetic distances among 14* Orobanche cumana* populations and subpopulations (260 individuals). Primary groups identified with either the 1st versus 2nd axis plot or with the 1st versus 3rd axis plot are highlighted with solid boxes. Populations from Spain (prefix IASCum) are named with their complete name, and populations from Bulgaria (prefix CUMBUL) are named with their number, without prefix.

For measuring differentiation between populations, pairwise *G*_ST_ values were computed (Table S2). No significant or very low (*G*_ST_ ≤ 0.01) differentiation was found for populations CUMBUL-8 and CUMBUL-9, collected on sunflower at close locations, CUMBUL-2 and CUMBUL-7, collected on* A. maritima* at near sites the same year, and CUMBUL-5_1 and CUMBUL-5_2, collected at the same location but on different wild hosts. Populations CUMBUL-1 and CUMBUL-7, which were collected at the same site but with a six-year difference, showed slightly higher *G*_ST_ values (0.107). The highest differentiation values (*G*_ST_ > 0.8) were found between the following three groups of populations: (i) IASCum-2, IASCum-3, and IASCum-4 collected on sunflower in Spain, (ii) CUMBUL-8 and CUMBUL-9 collected on sunflower in Bulgaria, and (iii) CUMBUL-3 and CUMBUL-4 collected on wild* A. maritima* in Bulgaria (Table S2). In principal coordinate analyses, the first three axes explained 39.4%, 26.6%, and 17.8%, respectively of the variation, producing five differentiated groups of populations: (i) IASCum-2 and IASCum-3, (ii) IASCum-4, (iii) CUMBUL-8 and CUMBUL-9, (iv) CUMBUL-3 and CUMBUL-4, and (v) the remaining seven populations, six of them collected on wild hosts in Bulgaria and one of them collected on sunflower in Bulgaria (Figure 3).

Bayesian-based analysis of the structure of the whole set of populations including those from Spain and Bulgaria with STRUCTURE revealed a close relationship among populations whatever their geographical origin, with an optimal *K* value of 2 (Figures S1 and S2). Secondary peaks were observed at *K* = 4 and 7 (Figure S1), and the standard deviation of Pr(*X*∣*K*) began to increase substantially at *K* values higher than these (Figure S1). Visualization of the cluster membership for *K* = 2 to *K* = 7 showed a general trend towards classification of populations IASCum-2, IASCum-3, IASCum-4, CUMBUL-3, CUMBUL-4, CUMBUL-8, and CUMBUL-9 within uniform pools, while the rest of the populations were included within mixed pools (Figure S2), assignments that recurred at all monitored levels of *K* (Figure S2).

**Figure 4:**
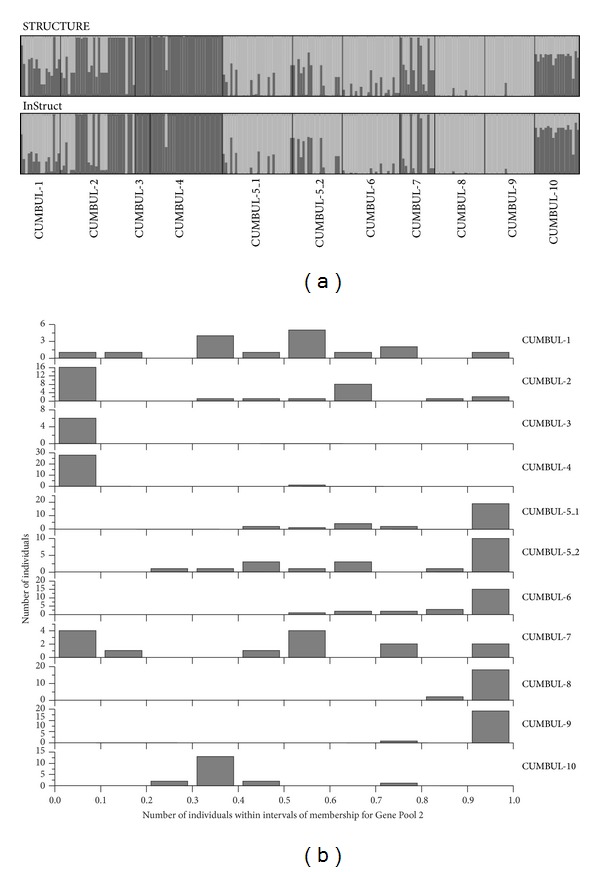
Results from STRUCTURE and InStruct analyses: (a) population structure obtained from STRUCTURE and InStruct analyses of eleven Bulgarian* Orobanche cumana* populations, with each individual being represented by a single vertical bar divided into two shades. Each shade represents one gene pool (*K*) and the length of the shaded segment shows the individual's estimated proportion of membership in that cluster and (b) number of* O. cumana* individuals from each Bulgarian population within intervals of membership for Gene Pool 2 in the STRUCTURE analyses.

A more detailed analysis of population structure including only Bulgarian populations was carried out. STRUCTURE analyses indicated the existence of two (*K* = 2; Figure S3) major genetic groups, mainly represented by populations CUMBUL-3 and CUMBUL-4 on one hand (Gene Pool 1), and CUMBUL-8 and CUMBUL-9 on the other hand (Gene Pool 2) (Table 3; Figure 4(a)). The remaining seven populations were categorized in-between these two groups, although the average proportion of membership was shifted towards Gene Pool 1 for populations CUMBUL-2 and CUMBUL-10, whereas populations CUMBUL-5_1, CUMBUL-5_2, and CUMBUL-6 were clearly shifted towards Gene Pool 2 (Table 3; Figure 1). When the membership value of each individual for each population was analyzed in detail, it was shown that an important number of individuals from populations CUMBUL-5_1, CUMBUL-5_2, and CUMBUL-6 [19 individuals out of 28 (67.9%), 10 out of 20 (50%), and 15 out of 23 (65.2%), resp.] showed a high (>0.90) membership value for Gene Pool 2 (Figure 4(b)). Classifications of individuals at *K* = 2 by the algorithms of STRUCTURE and InStruct were very similar qualitatively (Figure 4(a)). Within-cluster selfing rates estimated from InStruct analyses were very high (on average, 0.947 for Gene Pool 1 and 0.951 for Gene Pool 2).

Different AMOVA analyses were carried out within the* O. cumana* populations collected in Bulgaria. First, AMOVA analyses were conducted on populations collected on wild hosts. When no population structure was considered, 53.6% of the genetic variance was attributable to differences among populations, while the remaining 46.4% was due to differences within populations (Table 4). When populations were structured according to clustering results, differences among groups accounted for 50.4% of the total variance, while differences among populations of each group only accounted for 17.6% (Table 4). When populations collected on sunflower were added to the model, no significant structuring according to the ecological status of the populations was detected (Table 4). Structured analysis based on clustering groups produced similar results to the analysis of populations collected on wild hosts alone; that is, variation among groups accounted for 42.0% of total variation, while variation among populations at each group accounted for 25.1% (Table 4).

### 3.2. Parasitization Ability and Virulence on Sunflower

A first experiment demonstrated that* O. cumana* populations CUMBUL-1, CUMBUL-2, CUMBUL-4, and CUMBUL-5_1, collected on wild hosts, had the ability to parasitize sunflower lines B117 and B206, with no resistance genes, though some differences between populations were observed (Table 5). On B117, populations CUMBUL-1 and CUMBUL-5_1 produced similar number of shoots per sunflower plant to the control population OC-88, while CUMBUL-2 produced around four times more shoots per plant and CUMBUL-4 produced about half of shoots per plant than the control. On B206, both CUMBUL-1 and CUMBUL-2 yielded more shoots per plant than the control, while CUMBUL-4 produced less shoots per plant than the control (Table 5). In a second experiment the virulence of the populations collected on wild hosts, together with Bulgarian populations collected on sunflower, was evaluated on sunflower lines with varying degrees of genetic resistance. On sunflower line J8281, resistant to* O. cumana* race B, the number of shoots per sunflower plant did not differ significantly between Bulgarian* O. cumana* populations collected on wild hosts and those collected on sunflower (Table 6). The results were similar on sunflower line AC03-1589, resistant to race C, except for a significantly higher number of shoots in population CUMBUL-5_1. Similarly, the only wild population evaluated on line S1358 resistant to race D (CUMBUL-2) did not differ from the Bulgarian populations collected on sunflower. When the populations were tested on sunflower line P-1380, resistant to race E, only population CUMBUL-5_1 produced a few number of shoots per plant, whereas neither the other populations collected on wild species nor the Bulgarian populations collected on sunflower did possess the ability to parasitize P-1380. None of the populations parasitized on race-F resistant line P96 (Table 6).

## 4. Discussion

The genetic structure of* O. cumana* populations analyzed in this study was not determined by the fact that the populations were collected on wild or cultivated hosts. This was an unexpected result, since, within a number of largely self-pollinated parasitic plant species, host specificity has been found as a mechanism of accelerating isolation and subsequently genetic divergence among populations, for example, in* Orobanche minor* Sm. [52–54],* Striga asiatica* [23], and* S. gesnerioides* [24, 25]. Conversely, Vaz Patto et al. [20] studied the genetic structure of five Moroccan* O. foetida* populations, four of them parasitizing wild plants (*Scorpiurus muricatus* L. and* Ornithopus sativus* Brot.) and another one parasitizing cultivated vetch. The authors found that the vetch-parasitizing population was closer to the three populations parasitizing* S. muricatus*, while the population collected on* O. sativus* was the most genetically divergent. This suggested that parasitization of wild or cultivated hosts was not among the main factors determining genetic differences between these populations. Since host specificity in* Orobanche* spp. is mainly determined by induction of seed germination by specific chemical stimulants exuded by the host root [55], host-induced selection is expected to have an impact on very small portions of the genome, probably even at a single locus by modifying the binding site of the stimulant receptor [56]. Such limited genetic modifications, despite having a huge phenotypic impact, might not be detected with overall genome scans such as the one carried out in this research, while the rest of the genome is predominantly shaped by other evolutionary sources, namely, recombination and migration [29].

Nevertheless, an important observation in this study was that the genetic structure of wild* O. cumana* populations reflected introgressions from weedy populations parasitizing sunflower. This was shown not only by the analysis of population structure, but also by similar levels of virulence on sunflower of weedy and wild* O. cumana* populations. To the best of our knowledge, this is the first study on molecular diversity and virulence on sunflower of* O. cumana* populations parasitizing wild hosts. Previous studies focusing exclusively on weedy populations have shown the existence of several gene pools in this species, with low genetic diversity within each gene pool [27, 28, 57–59]. Gagne et al. [27] identified two gene pools, one of them comprising populations from eastern Europe (Romania, Bulgaria, and Turkey) and another one including populations from southern Spain. Studies on Spanish populations identified two well-separated gene pools, one of them in the south (Guadalquivir Valley) and another one in the central area (Cuenca Province) [28, 58, 59]. The study of Spanish populations [28] revealed that, although intrapopulation genetic diversity was in general extremely low, some populations showed larger diversity, which was hypothesized to be produced by genetic recombination between individuals from both gene pools. In the present research, two contrasting gene pools were identified in Bulgaria, one of them best represented by weedy populations from the central area (CUMBUL-8 and CUMBUL-9), and another one represented by wild populations from the eastern coast (CUMBUL-3 and CUMBUL-4), which showed in all cases low intrapopulation diversity. The fact that some wild populations had higher genetic diversity values and contained individuals that exhibited membership values very close to a weedy gene pool (>0.90) suggested the existence of genetic flow between both gene pools, which could be attributed to cross fertilization and/or seed movement. It is important to note that in the Black Sea coast of Bulgaria weedy and wild* O. cumana* populations coexist at short distances. The existence of cross fertilization within this species has been demonstrated in controlled experiments at a local scale [30] as well as in the molecular evaluation of field-collected weedy populations, where heterozygous individuals for unique alleles of different gene pools have been identified [28]. In relation to gene flow through seed dispersal,* Orobanche* seeds are easily dispersed by water, wind, and animals. Individual broomrape plants produce an impressive number of seeds from 50,000 to 500,000 [1] that maintain their viability in the soil for up to 20 years [11]. These seeds are of near-microscopic size, from 250 to 380 *μ*m long and from 150 to 240 *μ*m wide, with a weight from 1.0 to 2.5 *μ*g and are considered as “dust-seeds” [11, 15, 60, 61]. These factors are regarded as adaptations for being an obligate parasite, in order to be dispersed through vegetation so as to be as close as possible to the host plant and increasing the probability of finding an appropriate host [61]. Additionally, at a landscape scale,* Orobanche cumana* seed dispersion is highly influenced by human-derived agricultural and cultivation practices, as well as crop-seed trade and the use of contaminated sunflower seed stocks [15, 29], which might overpass spatial distances or barriers to gene flow common in natural ecosystems.

Wild and cultivated host plants represent different habitats for parasitic plants, especially when cultivated plants carry qualitative resistance genes, as is the case of the sunflower-*O. cumana* system [16]. The use of cultivars expressing vertical resistance mechanisms has contributed to a rapid development of* O. cumana* physiological races in most cultivation areas on the Old World, including Bulgaria [3, 19], which may explain why weedy populations of* O. cumana* generally show low genetic diversity, since new physiological races most likely evolve from single mutations events [62]. This is in general agreement with reports on plant pathogen-host interactions [22, 63, 64]. For parasitic plant-host interactions, higher intrapopulation variability was reported in a* Striga gesnerioides* population parasitizing the wild legume* Indigofera hirsuta* L. when compared to populations growing on cultivated cowpea [25]. Another study identified genetic diversity differences for a population of* S. hermonthica* grown on rice accessions of varying resistance to* Striga*, with the lowest diversity corresponding to a highly resistant rice accession [65]. This was not exactly the case of the present study, since we found genetic diversity values in wild populations similar or even lower than those reported in weedy* O. cumana* recombinant populations [28]. This could be explained on the basis of the existence of introgression from weedy populations into wild* O. cumana* populations. Studies in nonparasitic plant species, for example, in rice, have shown that introgression from cultivated species can considerably shape genetic diversity of wild populations [66, 67].

The study of* O. cumana* populations in their natural habitat provided new data about its breeding system. A clear heterozygote deficiency similar to that observed in populations parasitizing sunflower, deviation of genotypic frequencies at most loci from Hardy-Weinberg equilibrium, high inbreeding and selfing rate values, and the relatively low levels of genetic variation within populations coupled with substantial differences among populations, supported that wild populations of this species show a high degree of self-pollination, as reported previously for* O. cumana* populations parasitizing sunflower [27], and for other predominantly self-pollinating broomrape species such as* Phelipanche ramosa* (L.) Pomel [68].

*Orobanche* spp. differ for host specificity. Within this genus,* O. cumana* is one of species with the narrowest range of host plants. In the wild, it mainly parasitizes* Artemisia* spp. [4], whereas sunflower is the only crop in which* O. cumana* occurs as a parasitic weed [2].* Orobanche cumana* belongs to the native flora of Bulgaria, where it parasitizes wild species of the Asteraceae, mainly* A. maritima* [8]. Conversely, the genus* Helianthus* is from North American origin [69]. The first report of* O. cumana* parasitization on sunflower dates back to the 1890s in Russia [11] and to 1935 in Bulgaria [12]. It is unknown whether* O. cumana* possesses natural ability to parasitize sunflower or this ability arose in particular genotypes following mutation [70]. The possibility that* O. cumana* possesses natural ability to parasitize sunflower cannot be discarded, since molecules of the same nature to those involved in* O. cumana* stimulation of germination by sunflower root exudates occur commonly in plant organs of Asteraceae species [55]. The results of the present research did not shed light on this aspect, since both the population structure analysis as well as the virulence study indicated that the wild populations used in the study contain introgressions from weedy populations. The existence of genetic flow between* O. cumana* populations parasitizing sunflower and those parasitizing wild species opens up an interesting field of research on how increasing virulence in weedy populations observed in recent years in Bulgaria [19] may influence the parasitization ability of* O. cumana* on wild species and on how genetic variability of wild populations may favour the ability of weedy populations to overcome sunflower resistance mechanisms.

## Supplementary Material

Table S1: Characteristics (the expected band size, number of alleles, observed and expected heterozygosity, and *F*_ST_ values) of 15 microsatellite marker loci genotyped in 260 *Orobanche cumana* individuals from 14 populations and subpopulations.
Table S2: Values of the genetic distance coefficient *G*_ST_ calculated between all pairs of *O. cumana* populations collected on wild hosts (highlighted in bold) and on sunflower.
Figure S1: Description of the four steps (a), (b), (c), and (d) for the graphical method, according to Evanno [46], allowing the detection of the number of genetically homogeneous groups (K) that best fit the data in the STRUCTURE analyses including 14 *O. cumana* populations from Spain and Bulgaria. Figure S1(a), Mean of Pr(*X*∣*K*) (±SD) over 10 runs for each *K* value. Figure S1(b), Rate of change of the likelihood distribution. Figure S1(c), Absolute values of the second order rate of change of the likelihood distribution. Figure S1(d), DeltaK values with respect to K. Figure S1(e) presents detail of DeltaK values with respect to *K* from *K*=3 to *K*=14.
Figure S2: Results from STRUCTURE analyses including 14 *O. cumana* populations from Spain and Bulgaria (a) and (b). Figure S2(a) presents population structure with each individual being represented by a single vertical bar divided into colors, with each color representing one gene pool (*K*) and the length of the colored segment showing the individual's estimated proportion of membership in that cluster, and Figure S2(b) population structure in which each population is represented by its constituent clusters by averaging across individuals the membership coefficients for each cluster within each population.
Figure S3: Description of the four steps (a), (b), (c), and (d) for the graphical method, according to Evanno [46], allowing the detection of the number of genetically homogeneous groups (*K*) that best fit the data in the STRUCTURE analyses including 11 *O. cumana* populations from Bulgaria. Figure S3(a), Mean of Pr(*X*∣*K*) (±SD) over 10 runs for each *K* value. Figure S3(b), Rate of change of the likelihood distribution. Figure S3(c), Absolute values of the second order rate of change of the likelihood distribution. Figure S3(d), DeltaK values with respect to *K*.

## Figures and Tables

**Table 1:** Host species, collecting site, characteristics, and number of individuals analyzed for the studied *Orobanche cumana* populations.

Population	Host species	Collecting site	Region	Latitude, Longitude, Altitude	Year	*n*
*O. cumana* populations collected on wild hosts						
CUMBUL-1	*Artemisia maritima *	Bulgaria, Burgas, Atanasovsko Lake	South-Eastern Bulgaria	42°33′02.7′′N; 27°29′24′′E; 14 m	2006	16
CUMBUL-2	*Artemisia maritima *	Bulgaria, Burgas, Pomorie-Aheloj	South-Eastern Bulgaria	42°37′02.8′′N; 27°37′31.1′′E; 17 m	2006	30
CUMBUL-3	*Artemisia maritima *	Bulgaria, Kranevo	North-Eastern Bulgaria	43°20′05.6′′N; 28°3′41.9′′E; 112 m	2006	6
CUMBUL-4	*Artemisia maritima *	Bulgaria, Balchik	North-Eastern Bulgaria	43°24′36.9′′N; 28°9′23.5′′E; 21 m	2006	29
CUMBUL-5_1	*Anthemis arvensis *	Bulgaria, Kavarna, Gorun-Tyulenovo	North-Eastern Bulgaria	43°29′12.6′′N; 28°31′13.3′′E; 44 m	2006	28
CUMBUL-5_2	*Chamaemelum nobile *	Bulgaria, Kavarna, Gorun-Tyulenovo	North-Eastern Bulgaria	43°29′12.6′′N; 28°31′13.3′′E; 44 m	2006	20
CUMBUL-6	*Artemisia maritima *	Bulgaria, Burgas, Poda Protected Area	South-Eastern Bulgaria	42°26′35.91′′N; 27°27′58.64′′E; 7 m	2012	23
CUMBUL-7	*Artemisia maritima *	Bulgaria, Burgas, Atanasovsko Lake	South-Eastern Bulgaria	42°33′05.88′′N; 27°29′22.91′′E; 8 m	2012	14

*O. cumana *populations collected on sunflower						
CUMBUL-8	*Helianthus annuus *	Bulgaria, Sadovo	Central Bulgaria	42°07′13.49′′N; 24°54′53.40′′E; 156 m	2012	20
CUMBUL-9	*Helianthus annuus *	Bulgaria, Plodiv	Central Bulgaria	42°03′35.43′′N; 24°49′26.28′′E; 189 m	2012	20
CUMBUL-10	*Helianthus annuus *	Bulgaria, Balgarevo	North-Eastern Bulgaria	43°24′58.14′′N; 28°26′43.83′′E; 81 m	2012	18
IASCum-2	*Helianthus annuus *	Spain, Sevilla, Écija	Southern Spain	37°34′24′′N; 5°8′45′′W; 181 m	2008	12
IASCum-3	*Helianthus annuus *	Spain, Sevilla, Osuna	Southern Spain	37°15′19′′N; 5°3′49′′W; 304 m	2008	12
IASCum-4	*Helianthus annuus *	Spain, Cuenca, Montalbo	Central Spain	39°51′03′′N; 02°39′54′′W; 838 m	2008	12

*n*, final studied sample size [including a number of plants excluded from the analysis because of lack of amplification, belonging to each of the populations CUMBUL-1 (four plants excluded), CUMBUL-4 (one plant), CUMBUL-5_1 (two plants), CUMBUL-5_2 (two plants), CUMBUL-6 (three plants), and CUMBUL-10 (two plants)].

**Table 2:** Genetic diversity parameters of *Orobanche cumana* populations from Bulgaria collected on wild hosts and on sunflower (prefix CUMBUL-) and from Spain collected on sunflower (prefix IASCum-).

Population	P	Na (±SE)	Na ≥ 5% (±SE)	Ne (±SE)	Npa (±SE)	Ho (±SE)	He (±SE)	I (±SE)	Pairwise differences	Genotypic richness		F_is_ (±SE)	S
										*G*	*R*		
*O. cumana* populations collected on wild hosts													
CUMBUL-1	66.7	1.733 (0.15)	1.667 (0.13)	1.391 (0.10)	0.000 (0.00)	0.004 (0.01)	0.229 (0.05)	0.349 (0.08)	3.111 (1.66)	7	0.40	0.984 (0.01)	0.992
CUMBUL-2	86.9	2.000 (0.14)	1.933 (0.15)	1.521 (0.10)	0.000 (0.00)	0.000 (0.00)	0.297 (0.05)	0.458 (0.07)	4.393 (2.20)	8	0.24	1.000 (0.00)	1.000
CUMBUL-3	0.0	1.000 (0.00)	1.000 (0.00)	1.000 (0.00)	0.000 (0.00)	0.000 (0.00)	0.000 (0.00)	0.000 (0.00)	0.000 (0.00)	1	0.00	—	—
CUMBUL-4	53.3	1.600 (0.16)	1.333 (0.13)	1.145 (0.05)	0.067 (0.07)	0.000 (0.00)	0.105 (0.03)	0.184 (0.06)	1.595 (0.96)	6	0.18	1.000 (0.00)	1.000
CUMBUL-5_1	80.0	2.000 (0.17)	1.867 (0.16)	1.250 (0.07)	0.000 (0.00)	0.032 (0.01)	0.171 (0.04)	0.306 (0.06)	2.449 (1.34)	11	0.37	0.776 (0.04)	0.874
CUMBUL-5_2	80.0	1.933 (0.15)	1.933 (0.15)	1.418 (0.10)	0.000 (0.00)	0.014 (0.01)	0.248 (0.05)	0.400 (0.07)	3.597 (1.86)	7	0.32	0.920 (0.04)	0.958
CUMBUL-6	73.3	1.800 (0.14)	1.667 (0.16)	1.294 (0.10)	0.000 (0.00)	0.003 (0.01)	0.181 (0.05)	0.300 (0.07)	2.629 (1.43)	10	0.41	0.968 (0.03)	0.984
CUMBUL-7	73.3	1.800 (0.14)	1.800 (0.14)	1.467 (0.12)	0.000 (0.00)	0.010 (0.01)	0.258 (0.05)	0.398 (0.08)	3.947 (2.04)	7	0.46	0.975 (0.01)	0.987
*Mean *	64.2	1.733 (0.06)	1.650 (0.12)	1.311 (0.03)	0.008 (0.01)	0.008 (0.002)	0.186 (0.02)	0.299 (0.03)	2.715 (0.05)	7.1	0.30	0.946	0.971

*O. cumana* populations collected on sunflower													
CUMBUL-8	40.0	1.400 (0.13)	1.133 (0.09)	1.039 (0.02)	0.000 (0.00)	0.021 (0.01)	0.034 (0.01)	0.071 (0.03)	0.446 (0.41)	3	0.11	0.194 (0.10)	0.325
CUMBUL-9	13.3	1.133 (0.09)	1.133 (0.09)	1.014 (0.01)	0.000 (0.00)	0.000 (0.00)	0.013 (0.01)	0.026 (0.02)	0.195 (0.25)	2	0.05	1.000 (0.00)	1.000
CUMBUL-10	46.7	1.467 (0.13)	1.467 (0.13)	1.175 (0.06)	0.000 (0.00)	0.015 (0.01)	0.123 (0.04)	0.201 (0.06)	1.825 (1.07)	8	0.41	0.915 (0.04)	0.956
*Mean-Bulgaria *	33.3	1.333 (0.10)	1.244 (0.11)	1.076 (0.05)	0.000 (0.00)	0.012 (0.01)	0.057 (0.03)	0.099 (0.05)	0.822 (0.51)	4.3	0.19	0.703	0.760
IASCum-2	0.0	1.000 (0.00)	1.000 (0.00)	1.000 (0.00)	0.000 (0.00)	0.000 (0.00)	0.000 (0.00)	0.000 (0.00)	0.000 (0.00)	1	0.00	—	—
IASCum-3	0.0	1.000 (0.00)	1.000 (0.00)	1.000 (0.00)	0.000 (0.00)	0.000 (0.00)	0.000 (0.00)	0.000 (0.00)	0.000 (0.00)	1	0.00	—	—
IASCum-4	6.7	1.067 (0.07)	1.067 (0.07)	1.026 (0.03)	0.000 (0.00)	0.000 (0.00)	0.019 (0.02)	0.030 (0.03)	0.290 (0.32)	2	0.09	1.000 (0.02)	1.000
*Mean-Spain *	2.2	1.022 (0.02)	1.022 (0.02)	1.009 (0.01)	0.000 (0.00)	0.000 (0.00)	0.006 (0.01)	0.010 (0.01)	0.097 (0.10)	0.7	0.03		

*P*: percentage of polymorphic loci, Na: average observed allele number, Na > 5%: number of different alleles with a frequency ≥ 5%, Ne: number of effective alleles, Npa: number of private alleles unique to a single population, Ho: observed heterozygosity, He: expected heterozygosity, *I*: Shannon's diversity index, Pairwise differences: mean number of pairwise differences between individuals within each population (±SD), *G*: number of distinct multilocus genotypes (MLGs), *R*: genotypic richness; *F*_is_: fixation index, and *S*: selfing rate.

**Table 3:** Proportion of membership of each Bulgarian *Orobanche cumana* population in inferred STRUCTURE groups for *K* = 2. Populations collected on wild hosts are highlighted in bold.

Population	Genetic group 1	Genetic group 2
**CUMBUL-1**	0.491	0.509
**CUMBUL-2**	0.677	0.323
**CUMBUL-3**	0.984	0.016
**CUMBUL-4**	0.969	0.031
**CUMBUL-5_1**	0.138	0.862
**CUMBUL-5_2**	0.239	0.76
**CUMBUL-6**	0.104	0.896
**CUMBUL-7**	0.541	0.459
CUMBUL-8	0.023	0.977
CUMBUL-9	0.022	0.978
CUMBUL-10	0.647	0.352

**Table 4:** Analysis of molecular variance (AMOVA) of *Orobanche cumana* populations from Bulgaria.

Hierarchical structure and source of variation	AMOVA statistics				*F*-statistics^a^	*P* value
	df	Sum of squares	Variance components	% Variance		
Bulgarian populations collected on wild hosts (8 populations; 166 individuals)						
Not structured						
Among populations	7	491.05	1.69	53.64	*F*_ST_ = 0.54	<0.001
Within populations/group	324	473.19	1.46	46.36		
Structured based on gene pools^b^						
Among groups	1	294.36	2.29	50.37	*F*_CT_ = 0.50	0.032
Among populations/group	6	196.69	0.80	17.56	*F*_SC_ = 0.35	<0.001
Within populations/group	324	473.19	1.46	32.07	*F*_ST_ = 0.68	<0.001

Total of Bulgarian populations (wild and cultivated host) (11 populations; 224 individuals)						
Not structured						
Among populations	10	713.97	1.74	59.54	*F*_ST_ = 0.60	<0.001
Within populations/group	437	517.64	1.18	40.46		
Structured based on ecological status^c^						
Among groups	1	93.81	0.14	4.55	*F*_CT_ = 0.05	0.234
Among populations/group	9	620.16	1.68	56.05	*F*_SC_ = 0.59	<0.001
Within populations/group	437	517.64	1.18	39.40	*F*_ST_ = 0.61	<0.001
Structured based on gene pools^d^						
Among groups	2	423.05	1.51	42.01	*F*_CT_ = 0.42	0.002
Among populations/group	8	290.91	0.90	25.05	*F*_SC_ = 0.43	<0.001
Within populations/group	437	517.64	1.18	32.94	*F*_ST_ = 0.67	<0.001

^a^ *F*-statistics represents differentiation among groups (*F*_CT_), among populations within groups (*F*_SC_), and among populations within the whole population (*F*_ST_).

^b^The gene pools defined with clustering analyses comprised (i) populations CUMBUL-3 and -4 and (ii) populations CUMBUL-1, -2, -5_1, -5_2, -6, and -7.

^c^The structured groups based on the ecological status were (i) wild hosts (populations CUMBUL-1, -2, -3, -4, -5_1, -5_2, -6, and -7) and (ii) cultivated host (sunflower) (populations CUMBUL-8, -9, -10).

^d^The gene pools defined with clustering analyses were (i) populations CUMBUL-3, -4, (ii) populations CUMBUL-8 and -9, and (iii) populations CUMBUL-1, -2, -5_1, -5_2, -6, -7, and -10.

**Table 5:** Number of emerged *Orobanche cumana* shoots per sunflower plant (mean ± standard deviation) in the evaluation of *O. cumana* populations CUMBUL-1, CUMBUL-2, and CUMBUL-4, collected in Bulgaria on *Artemisia maritima*, CUMBUL-5_1, collected in Bulgaria on *Anthemis arvensis*, and control population OC-88, collected in Spain on cultivated sunflower, on two sunflower lines (B117 and B206) with no genetic resistance to *O. cumana*, conducted in pots in 2007^a^.

	B117^b^	B206^a^
CUMBUL-1	14.5 ± 9.9^b^	35.3 ± 13.1^c^
CUMBUL-2	39.5 ± 7.7^c^	36.3 ± 11.3^c^
CUMBUL-4	5.2 ± 4.7^a^	1.7 ± 1.0^a^
CUMBUL-5_1	11.5 ± 8.9^ab^	14.3 ± 4.5^b^
OC-88	10.3 ± 6.0^ab^	18.5 ± 7.2^b^

^a^Eight pots per each combination of sunflower cultivar and *O. cumana* population.

^b^Means with different letters for each sunflower cultivar differ significantly (*P* < 0.05).

**Table 6:** Number of emerged *Orobanche cumana* shoots per sunflower plant (mean ± standard deviation) in the evaluation of *O. cumana* populations CUMBUL-1, CUMBUL-2, and CUMBUL-4, collected in Bulgaria on *Artemisia maritima*, CUMBUL-5_1, collected in Bulgaria on *Anthemis arvensis*, OC-9, OC-11, and OC-13, collected in Bulgaria on cultivated sunflower, and OC-88, collected in Spain on cultivated sunflower, on six sunflower lines with different levels of genetic resistance, conducted in pots in 2008^a^. The *O. cumana* race to which each sunflower line is expected to be resistant (if any) is given in parenthesis.

	B117^b^	J8281 (B)	AC03-1589 (C)	S1358 (D)	P-1380 (E)	P96 (F)
CUMBUL-1	17.7 ± 6.3^bc^	2.6 ± 2.4^ab^	1.1 ± 1.2^a^	NE^c^	0^a^	0
CUMBUL-2	20.6 ± 3.2^c^	1.3 ± 1.7^ab^	0.5 ± 1.1^a^	2.0 ± 1.4^a^	0^a^	0
CUMBUL-4	10.9 ± 7.1^ab^	0.1 ± 0.4^a^	0.6 ± 0.7^a^	NE	0^a^	0
CUMBUL-5_1	12.6 ± 6.7^ab^	2.0 ± 1.1^ab^	5.1 ± 2.6^b^	NE	0.4 ± 0.7^a^	0
OC-9	13.8 ± 7.4^abc^	3.0 ± 2.5^ab^	0.5 ± 0.8^a^	0.8 ± 1.0^a^	0^a^	0
OC-11	8.6 ± 5.3^a^	2.5 ± 2.1^ab^	0.2 ± 0.4^a^	0.7 ± 0.8^a^	0^a^	0
OC-13	14.3 ± 7.9^abc^	4.1 ± 1.9^b^	1.1 ± 1.1^a^	1.2 ± 0.8^a^	0^a^	0
OC-88	9.9 ± 6.9^a^	16.6 ± 6.5^c^	1.6 ± 1.3^a^	1.2 ± 1.2^a^	6.9 ± 4.3^b^	0

^a^Eight pots per each combination of sunflower cultivar and *O. cumana* population.

^b^Means with different letters for each sunflower cultivar differ significantly (*P* < 0.05).

^c^NE = not evaluated.
