# Supplementary material for: The Genetic Structure of Wild Orobanche cumana Wallr. (Orobanchaceae) Populations in Eastern Bulgaria Reflects Introgressions from Weedy Populations
Source: ScientificWorldJournal. 2014 Jul 20;2014:150432. doi: 10.1155/2014/150432 (PMC4131075; doi:10.1155/2014/150432)
Supplement: Supplementary file 1 — Table S1: Characteristics (the expected band size, number of alleles, observed and expected heterozygosity, and F ST values) of 15 microsatellite marker loci genotyped in 260 Orobanche cumana individuals from 14 populations and subpopulations. Table S2: Values of the genetic distance coefficient G ST calculated between all pairs of O. cumana populations collected on wild hosts (highlighted in bold) and on sunflower. Figure S1: Description of the four steps (a), (b), (c), and (d) for the graphical method, according to Evanno [46], allowing the detection of the number of genetically homogeneous groups (K) that best fit the data in the STRUCTURE analyses including 14 O. cumana populations from Spain and Bulgaria. Figure S1(a), Mean of Pr(X∣K) (±SD) over 10 runs for each K value. Figure S1(b), Rate of change of the likelihood distribution. Figure S1(c), Absolute values of the second order rate of change of the likelihood distribution. Figure S1(d), DeltaK values with respect to K. Figure S1(e) presents detail of DeltaK values with respect to K from K=3 to K=14. Figure S2: Results from STRUCTURE analyses including 14 O. cumana populations from Spain and Bulgaria (a) and (b). Figure S2(a) presents population structure with each individual being represented by a single vertical bar divided into colors, with each color representing one gene pool (K) and the length of the colored segment showing the individual's estimated proportion of membership in that cluster, and Figure S2(b) population structure in which each population is represented by its constituent clusters by averaging across individuals the membership coefficients for each cluster within each population. Figure S3: Description of the four steps (a), (b), (c), and (d) for the graphical method, according to Evanno [46], allowing the detection of the number of genetically homogeneous groups (K) that best fit the data in the STRUCTURE analyses including 11 O. cumana populations from Bulgaria. Figure S3(a), Mean of Pr [file 150432.f1.pdf]

Figure S1: Description of the four steps (a), (b), (c), and (d), for the graphical method, according to Evanno [41], allowing the detection of the number of genetically homogeneous groups (*K*) that best fit the data in the STRUCTURE analyses including 14 *O. cumana* populations from Spain and Bulgaria. Figure S1(a), Mean of Pr(X|*K*) (±SD) over 10 runs for each *K* value. Figure S1(b), Rate of change of the likelihood distribution. Figure S1(c), Absolute values of the second order rate of change of the likelihood distribution. Figure S1(d), Delta*K* values with respect to *K*. Figure S1(e) presents detail of Delta*K* values with respect to *K* from K=3 to K=14

(a)

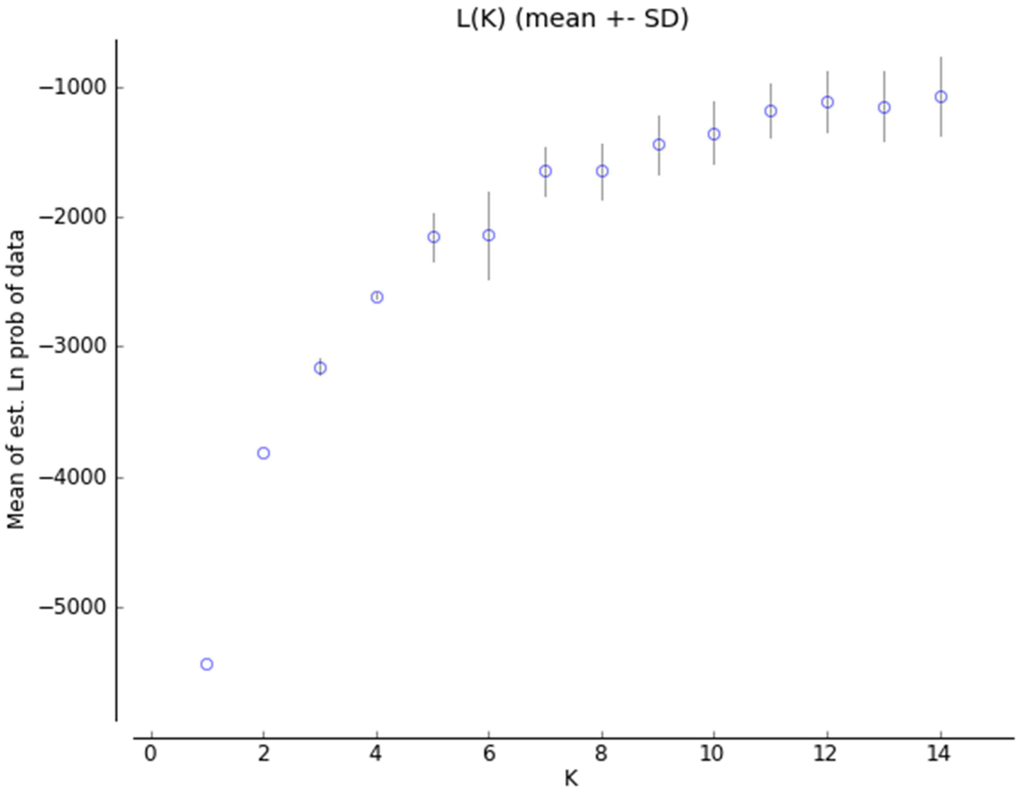

(b)

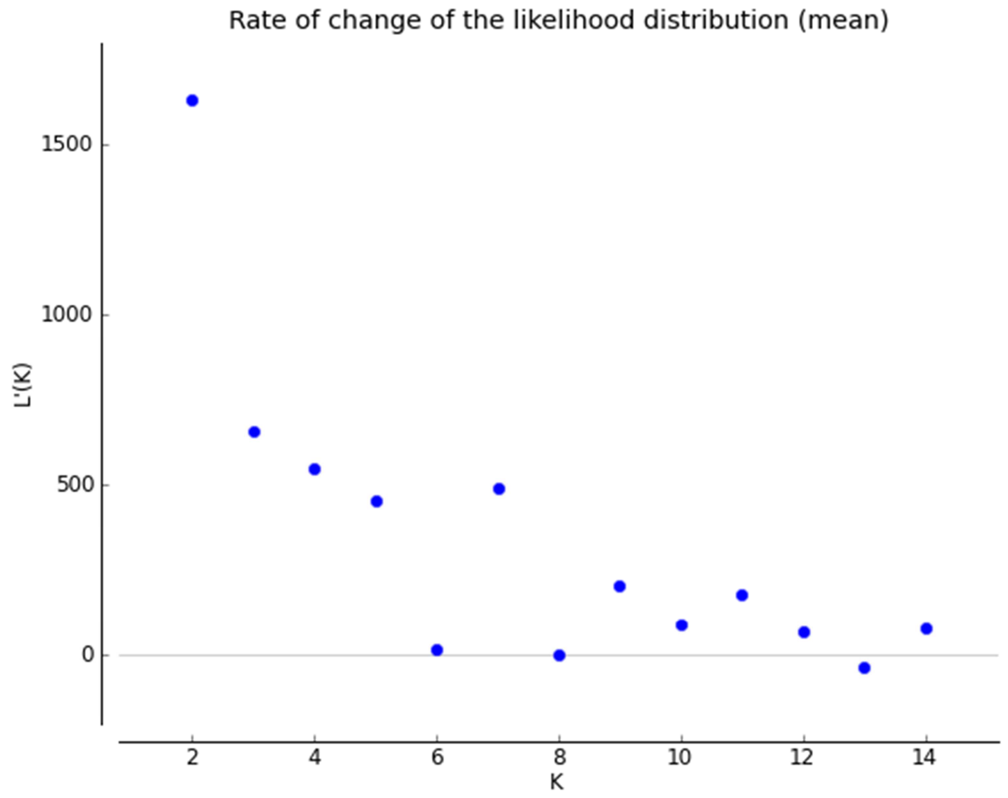

Rate of change of the likelihood distribution (mean)
L'(K)
1500
1000
500
0
2
4
6
8
10
12
14
K

(c)

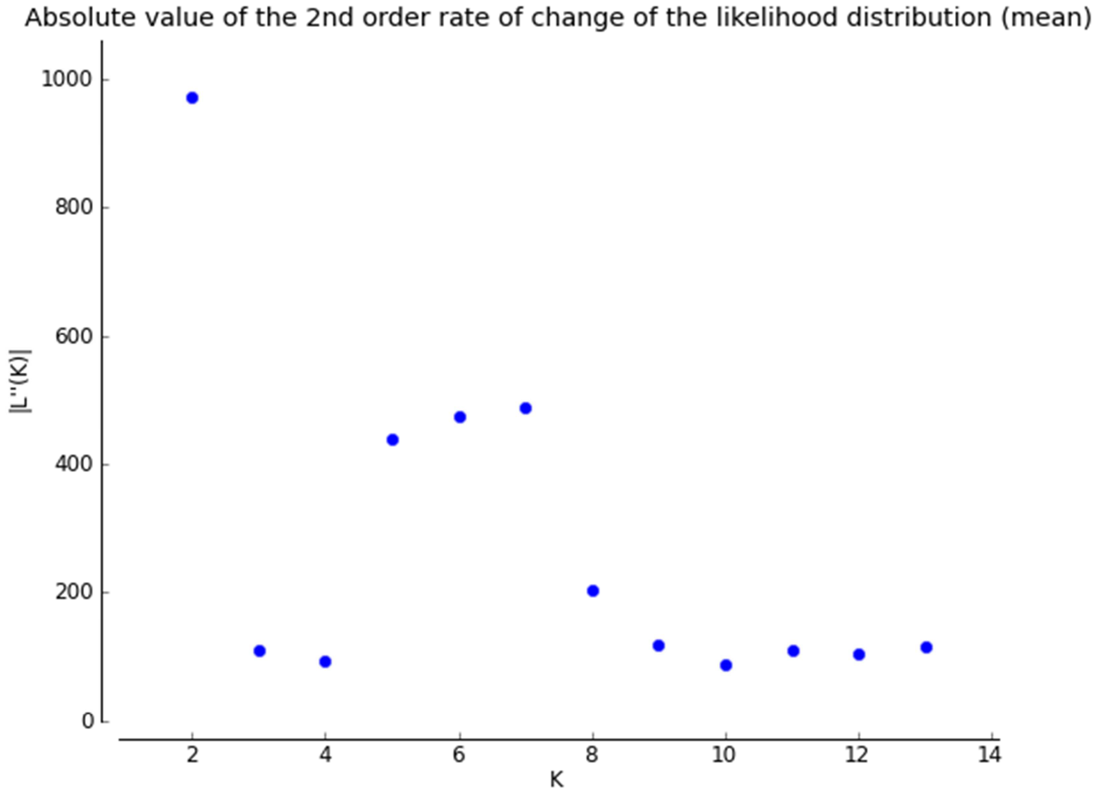

Absolute value of the 2nd order rate of change of the likelihood distribution (mean)
|L''(K)|
1000
800
600
400
200
0
2
4
6
8
10
12
14
K

(d)

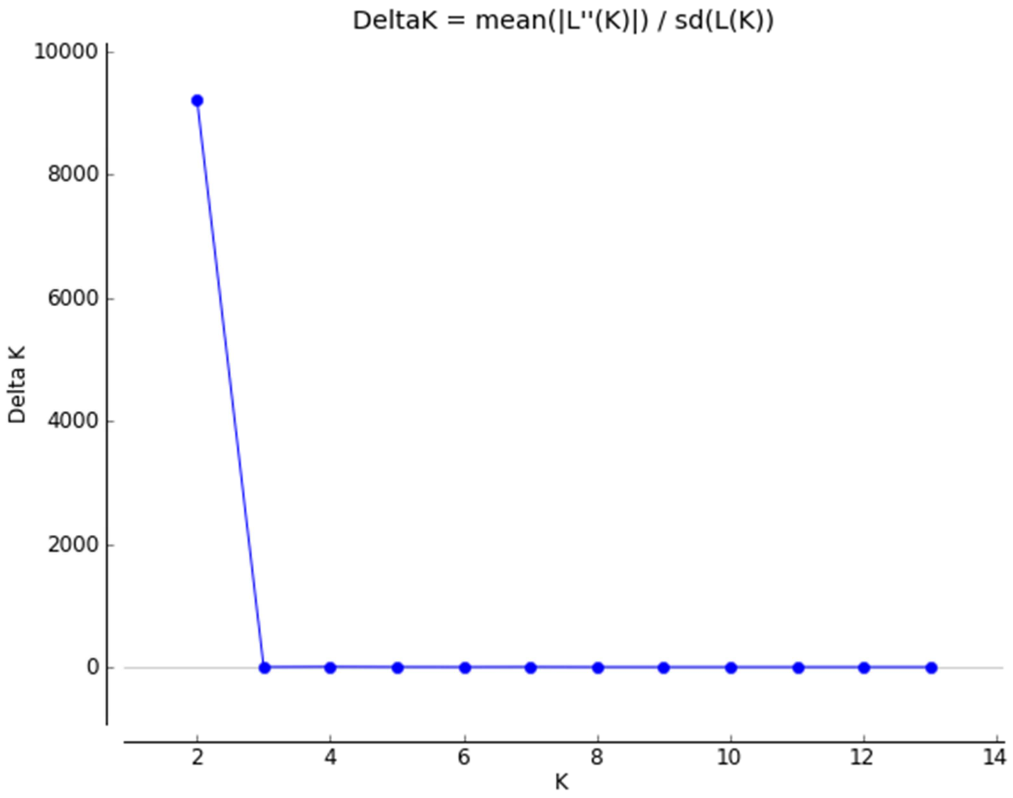
DeltaK = mean(|L''(K)|) / sd(L(K))
Delta K
10000
8000
6000
4000
2000
0
2
4
6
8
10
12
14
K

(e)

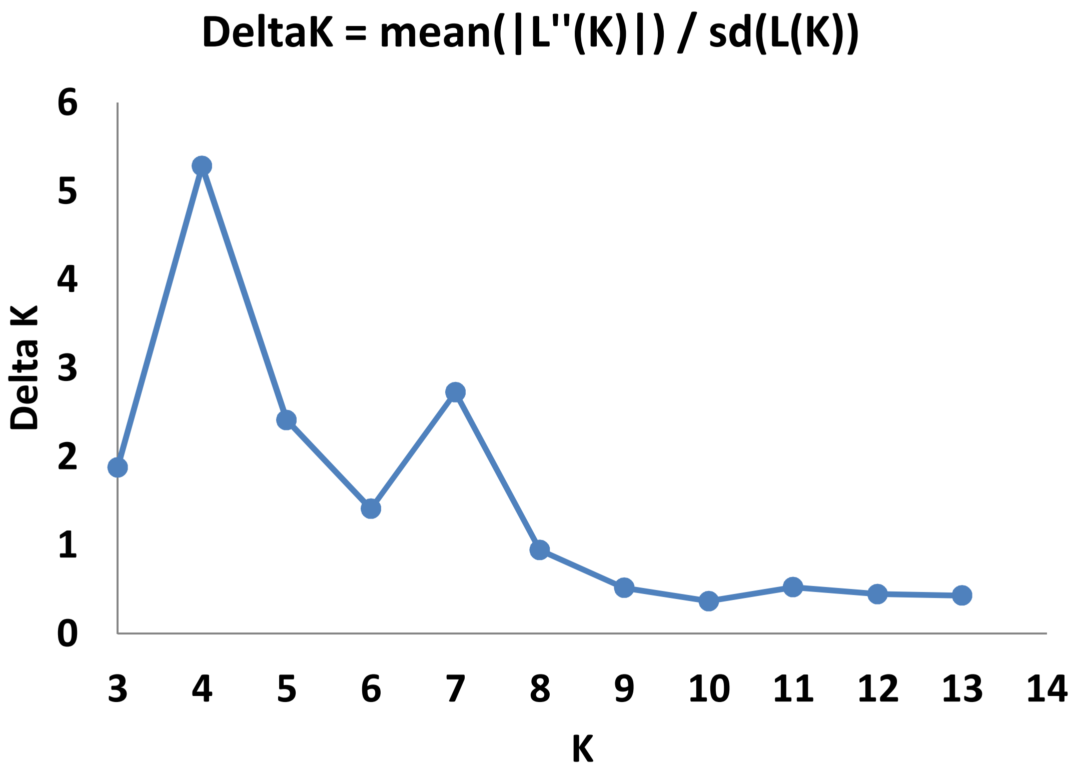
DeltaK = mean(|L''(K)|) / sd(L(K))
Delta K
6
5
4
3
2
1
0
3
4
5
6
7
8
9
10
11
12
13
14
K

Figure S2: Results from STRUCTURE analyses including 14 *O. cumana* populations from Spain and Bulgaria (a) and (b). Figure S2(a) presents population structure with each individual being represented by a single vertical bar divided into colors, with each color representing one gene pool (*K*) and the length of the colored segment showing the individual's estimated proportion of membership in that cluster, and Figure S2(b) population structure in which each population is represented by its constituent clusters by averaging across individuals the membership coefficients for each cluster within each population

(a)

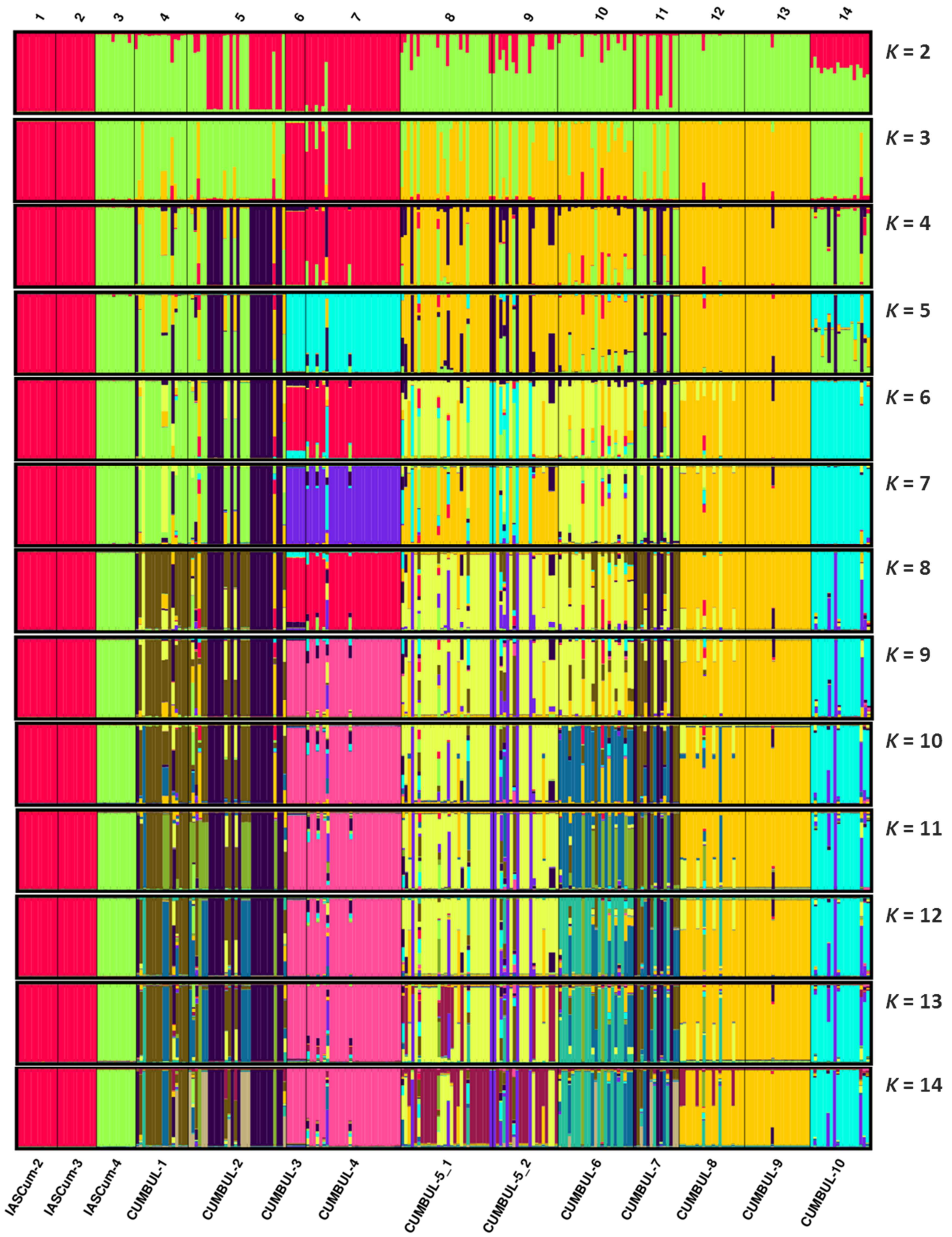

(b)

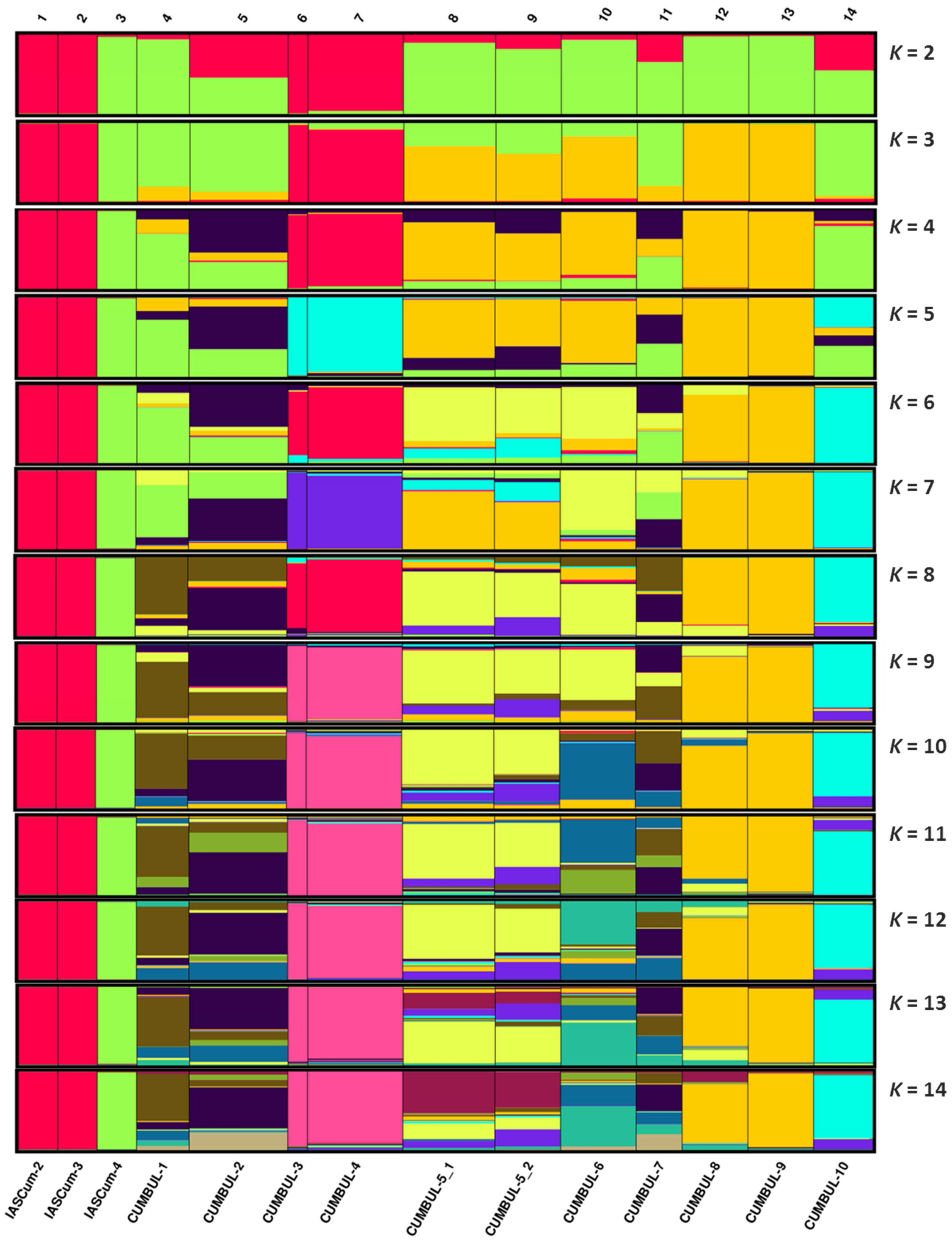
1
2
3
4
5
6
7
8
9
10
11
12
13
14
K = 2
K = 3
K = 4
K = 5
K = 6
K = 7
K = 8
K = 9
K = 10
K = 11
K = 12
K = 13
K = 14
IASCum-2
IASCum-3
IASCum-4
CUMBUL-1
CUMBUL-2
CUMBUL-3
CUMBUL-4
CUMBUL-5_1
CUMBUL-5_2
CUMBUL-6
CUMBUL-7
CUMBUL-8
CUMBUL-9
CUMBUL-10

Figure S3: Description of the four steps (a), (b), (c), and (d), for the graphical method, according to Evanno [41], allowing the detection of the number of genetically homogeneous groups (*K*) that best fit the data in the STRUCTURE analyses including 11 *O. cumana* populations from Bulgaria. Figure S3(a), Mean of Pr(X|*K*) (±SD) over 10 runs for each *K* value. Figure S3(b), Rate of change of the likelihood distribution. Figure S3(c), Absolute values of the second order rate of change of the likelihood distribution. Figure S3(d), Delta*K* values with respect to *K*

(a)

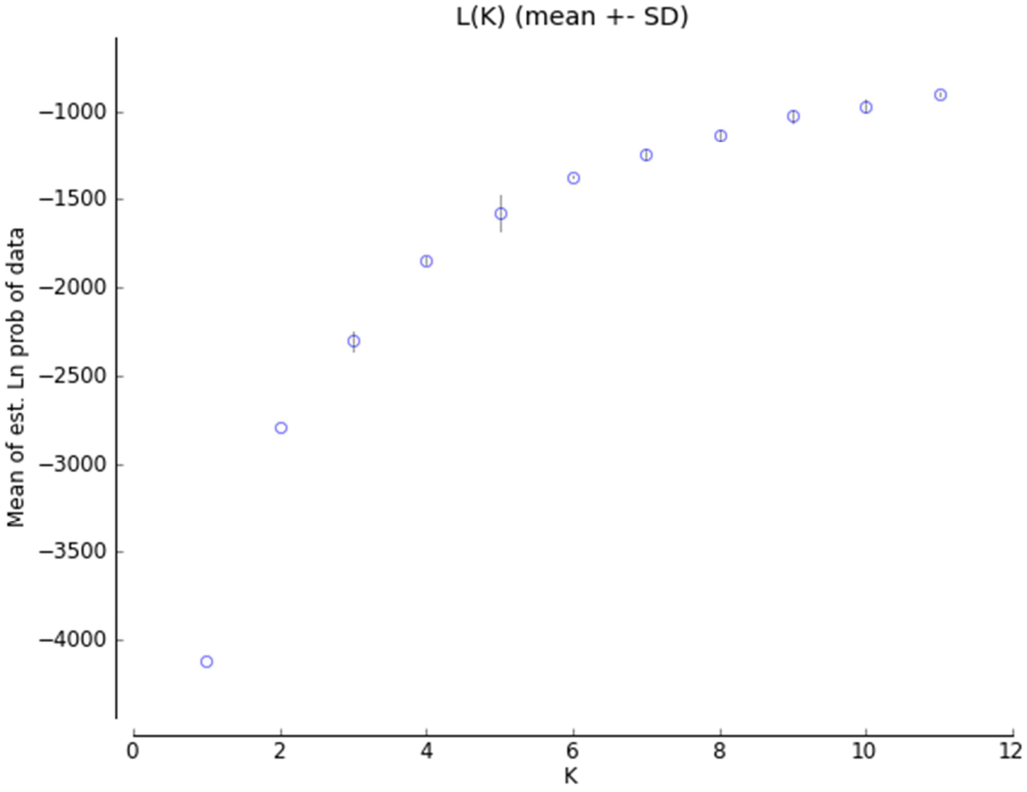

(b)

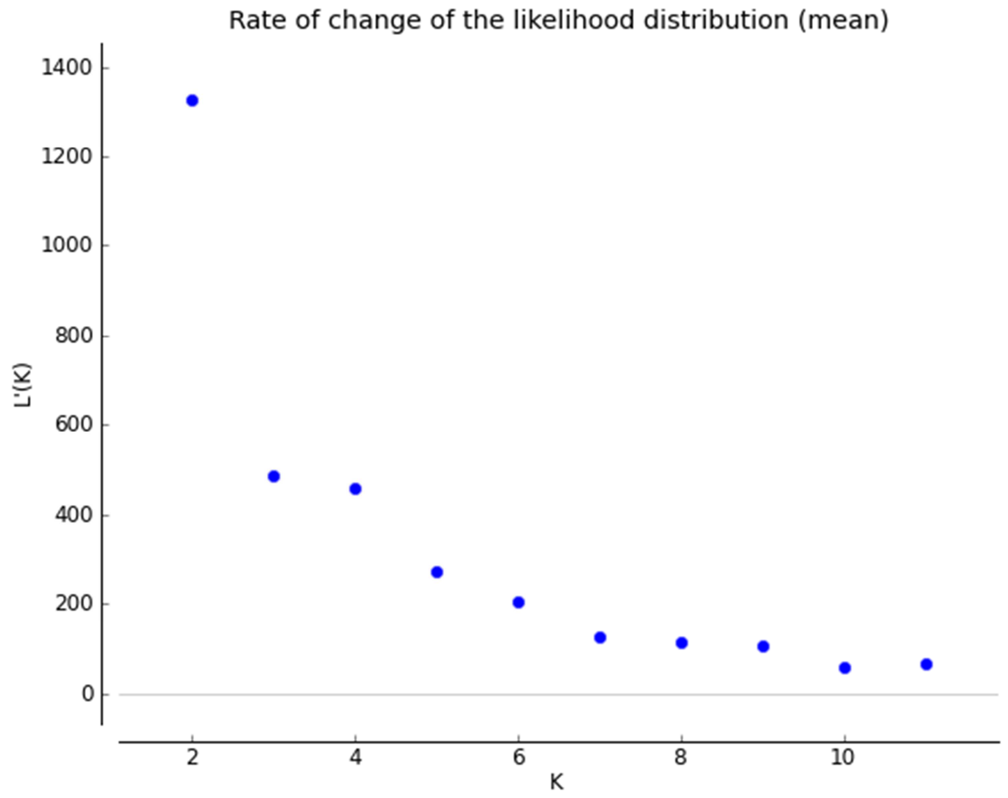
Rate of change of the likelihood distribution (mean)
L'(K)
1400
1200
1000
800
600
400
200
0
2
4
6
8
10
K

(c)

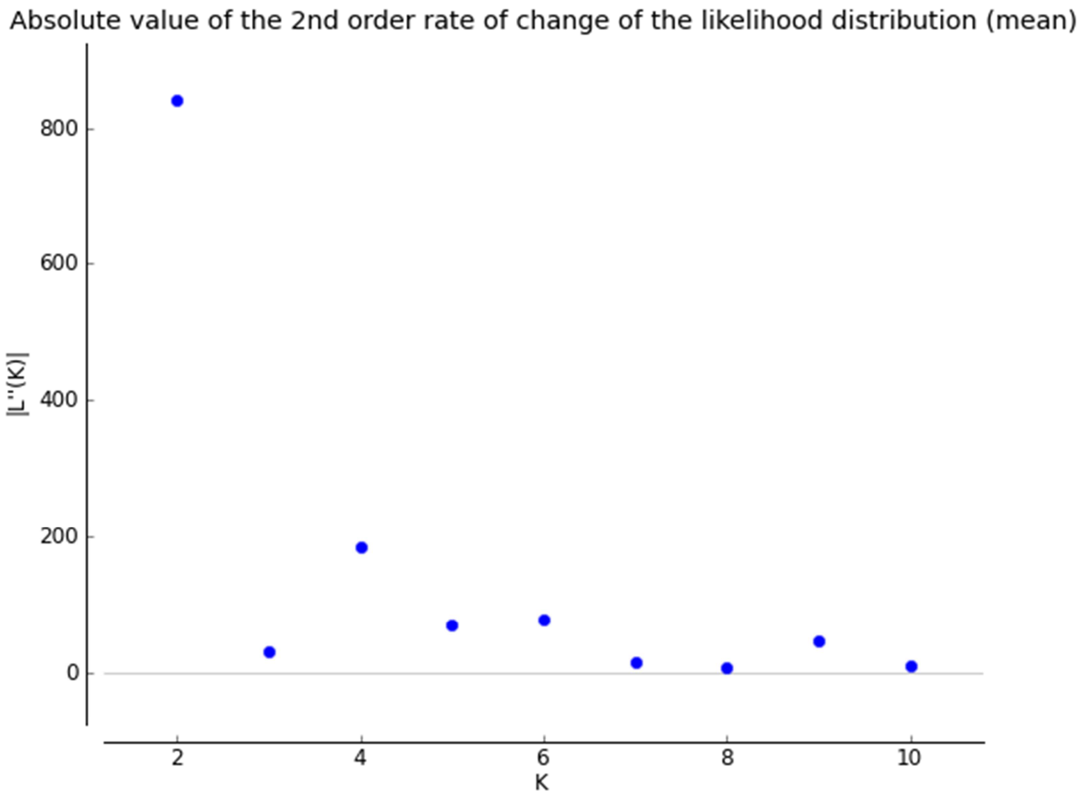
Absolute value of the 2nd order rate of change of the likelihood distribution (mean)
|L''(K)|
800
600
400
200
0
2
4
6
8
10
K

(d)

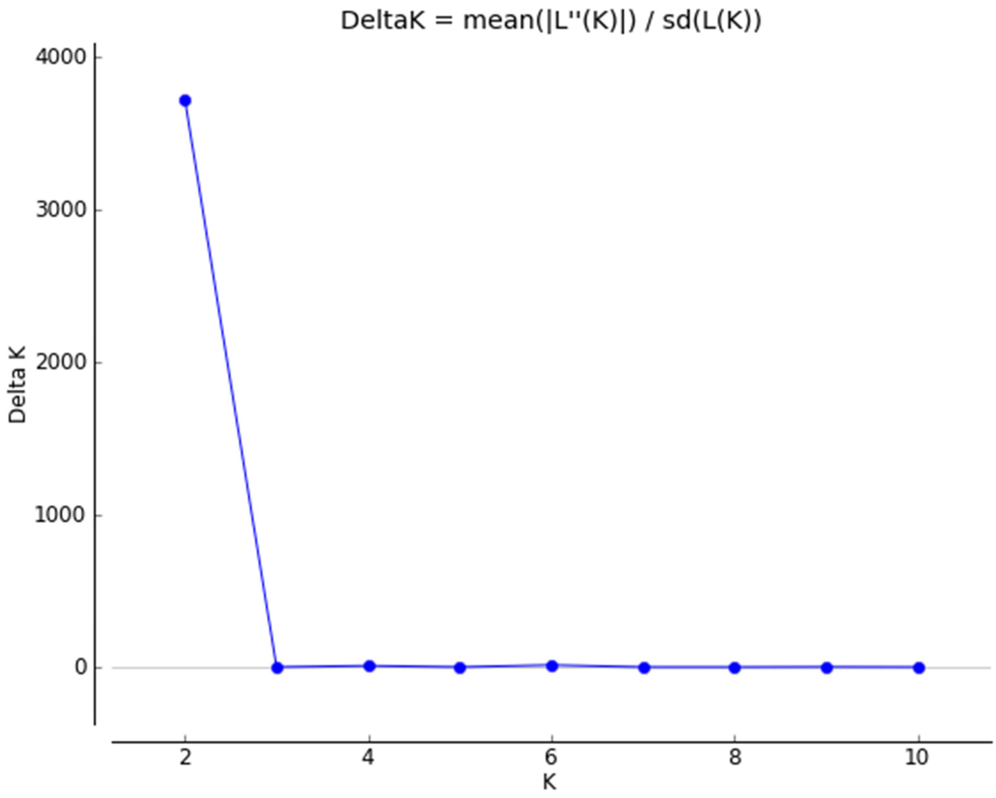

Table S1: Characteristics of 15 microsatellite loci genotyped in 260 *Orobanche cumana* individuals from 14 populations and subpopulations. Na: average observed allele number, Ho: observed heterozygosity, He: expected heterozygosity.

SSR marker	Allele size (bp)	Na (± SE)	Ho (± SE)	He (± SE)	F_{ST}
Ocum-052	108	1.643 (0.13)	0.011 (0.006)	0.148 (0.04)	0.761
Ocum-059	90	1.571 (0.14)	0.003 (0.003)	0.153 (0.05)	0.682
Ocum-070	96	1.357 (0.13)	0.006 (0.004)	0.095 (0.04)	0.813
Ocum-074	99	1.714 (0.22)	0.018 (0.008)	0.176 (0.06)	0.690
Ocum-075	110	1.357 (0.13)	0.000 (0.000)	0.076 (0.03)	0.806
Ocum-081	90	1.571 (0.17)	0.008 (0.005)	0.138 (0.05)	0.762
Ocum-087	134	1.500 (0.14)	0.000 (0.000)	0.091 (0.03)	0.847
Ocum-108	143	1.214 (0.11)	0.004 (0.004)	0.024 (0.01)	0.910
Ocum-122	241	1.429 (0.14)	0.006 (0.004)	0.115 (0.05)	0.566
Ocum-141	191	1.500 (0.14)	0.008 (0.008)	0.169 (0.05)	0.646
Ocum-160	136	1.643 (0.13)	0.018 (0.012)	0.187 (0.05)	0.564
Ocum-174	190	1.429 (0.14)	0.003 (0.003)	0.130 (0.05)	0.616
Ocum-196	192	1.929 (0.24)	0.008 (0.006)	0.187 (0.05)	0.699
Ocum-197	95	1.500 (0.20)	0.013 (0.007)	0.087 (0.04)	0.834
Ocum-206	119	1.071 (0.07)	0.000 (0.000)	0.020 (0.02)	0.924

Table S2: Values of the genetic distance coefficient G_{ST} calculated between all pairs of *Orobanche cumana* populations collected on wild hosts (highlighted in bold) and on sunflower.

	IASCum-2	IASCum-3	IASCum-4	**CUMBUL-1**	**CUMBUL-2**	**CUMBUL-3**	**CUMBUL-4**	**CUMBUL-5_1**	**CUMBUL-5_2**	**CUMBUL-6**	**CUMBUL-7**	CUMBUL-8	CUMBUL-9
IASCum-3	0,000^{ns}												
IASCum-4	0,980	0,980											
**CUMBUL-1**	0,710	0,710	0,490										
**CUMBUL-2**	0,575	0,575	0,533	0,159									
**CUMBUL-3**	1,000	1,000	0,972*	0,651	0,489								
**CUMBUL-4**	0,808	0,808	0,822	0,561	0,441	0,483							
**CUMBUL-5_1**	0,739	0,739	0,676	0,345	0,238	0,696	0,615						
**CUMBUL-5_2**	0,638	0,638	0,585	0,290	0,158	0,571	0,521	0,008^{ns}					
**CUMBUL-6**	0,751	0,751	0,627	0,234*	0,233	0,715	0,645	0,166	0,151				
**CUMBUL-7**	0,633	0,633	0,556	0,107*	0,008 ^{ns}	0,545	0,490	0,237	0,171	0,170			
CUMBUL-8	0,948	0,948	0,902	0,479	0,402	0,940	0,809	0,241	0,224	0,209	0,385		
CUMBUL-9	0,980	0,980	0,941	0,510	0,424	0,976	0,836	0,285	0,257	0,256	0,415	0,012*	
CUMBUL-10	0,825	0,825	0,738	0,456	0,344	0,697	0,561	0,433	0,331	0,487	0,371	0,677	0,721

All values were significant at $P = 0.001$ based on 999 random permutations of the data, with the exception of those indicated with the symbols *: significant at $P < 0.01$, and ns: Non-significant.
